# T Cells in Preterm Infants and the Influence of Milk Diet

**DOI:** 10.3389/fimmu.2020.01035

**Published:** 2020-06-02

**Authors:** Thomas Sproat, Rebecca Pamela Payne, Nicholas D. Embleton, Janet Berrington, Sophie Hambleton

**Affiliations:** ^1^Neonatal Intensive Care Unit, Royal Victoria Infirmary, Newcastle upon Tyne, United Kingdom; ^2^Immunity and Inflammation Theme, Translational and Clinical Research Institute, Newcastle University, Newcastle upon Tyne, United Kingdom; ^3^Population Health Science Institute, Newcastle University, Newcastle upon Tyne, United Kingdom

**Keywords:** T-lymphocytes, infant, premature, mucosal immunity, necrotizing enterocolitis, extremely premature

## Abstract

Preterm infants born before 32 weeks gestational age (GA) have high rates of late onset sepsis (LOS) and necrotizing enterocolitis (NEC) despite recent improvements in infection control and nutrition. Breast milk has a clear protective effect against both these outcomes likely due to multiple mechanisms which are not fully understood but may involve effects on both the infant's immune system and the developing gut microbiota. Congregating at the interface between the mucosal barrier and the microbiota, innate and adaptive T lymphocytes (T cells) participate in this interaction but few studies have explored their development after preterm delivery. We conducted a literature review of T cell development that focuses on fetal development, postnatal maturation and the influence of milk diet. The majority of circulating T cells in the preterm infant display a naïve phenotype but are still able to initiate functional responses similar to those seen in term infants. T cells from preterm infants display a skew toward a T-helper 2(T_h_2) phenotype and have an increased population of regulatory cells (T_reg_s). There are significant gaps in knowledge in this area, particularly in regards to innate-like T cells, but work is emerging: transcriptomics and mass cytometry are currently being used to map out T cell development, whilst microbiomic approaches may help improve understanding of events at mucosal surfaces. A rapid rise in organoid models will allow robust exploration of host-microbe interactions and may support the development of interventions that modulate T-cell responses for improved infant health.

## Introduction

Infants born prematurely, especially before 32 weeks gestational age (GA) are susceptible to diseases associated with invasive bacterial infections, specifically late onset sepsis (LOS) and necrotizing enterocolitis (NEC). These occur in around 30 and 6% of very preterm infants, respectively, according to recent data (1), most occur in the first 4 weeks of life, and both diseases are associated with changes in the gut microbiota (2, 3). Feeding preterm infants with their mother's breast milk has been shown to reduce the risk of NEC and LOS (4, 5). T lymphocytes (T cells) are an important component of the immune response to infection, however, this role is balanced against their emerging role in tissue regeneration and repair. The newborn, who is exposed to diverse microbes including many potential pathogens soon after birth, requires an appropriate T cell response to navigate the potentially conflicting requirements of growth, repair and infection control.

This review focuses on fetal T cell development, postnatal T cell maturation, and the potential for dietary modulation of gut mucosal T cells in the preterm environment. T cells of particular interest in the preterm infant comprise those with innate-like properties such as γδ T, invariant natural killer T (iNKT) and mucosa-associated invariant T (MAIT) cells, which have the capacity to deliver effector functions without prior clonal expansion. Among conventional TCRαβ-expressing T cells, regulatory T cells (T_reg_s) are known for their ability to mediate tolerance but also have emerging roles in tissue regeneration of potential relevance to NEC (Table 1).

**Table 1:** Summary of differences between peripheral blood T cell populations in preterm infants compared to term infants.

**T cell type**	**Description of role**	**Relative abundance (preterm compared to term infants, population as % of T cells)**	**Relative function (preterm compared to term infants)**	**Relevance of difference**	**References**
CD4+ T (T_h_)	Regulate immune response Help B cells produce immunoglobulin	↑↑	↑IL-5 ↓IFN-γ	Limited T_h_1 response limits response to intracellular infections	(6, 7)
T_reg_	Suppress immune response	↑↑	↑IL-10	Suppress immune response, leading to tolerogenic state (limiting inflammation)	(6, 8–10)
CD8+ T	Cytotoxic T cells	↔	Not known ↑IFN-γ^*^ ↑TNF-α^*^ ↓IL-2^*^	Potentially increased ability to respond to viral infections	(11, 12)
γδ T	Innate-like, predominantly mucosa-based range of cytotoxic functions	↓↓	↑IFN-γ ↑IL-10	Potentially compensates for decreased T_h_1 response.	(13–15)
iNKT	Innate-like, some roles similar to natural killer cells	Not known/↑^*^	Not known	Unclear if beneficial or detrimental	(16)
MAIT	Innate-like, cytotoxic and inflammatory functions	↓	Not known	Unclear if beneficial or detrimental	(17, 18)

* *Umbilical cord blood. ↑Increased; ↑↑Greatly increased; ↓Decreased; ↓↓Greatly decreased; ↔Similar*.

A challenge of studying T cells in preterm infants is that it is difficult to obtain suitable samples purely for research purposes. Umbilical cord blood is often used to represent the immune system of preterm infants, however a recent systems level analysis suggests it is more representative of the fetal state than the *ex-utero* infant (6). Therefore, where possible, we focus on studies using postnatal blood, but supplement these data with studies on umbilical cord blood, fetuses, and animal studies as necessary.

## Fetal T-Cell Development

Bone marrow-derived T progenitor cells enter the thymus at around 8 weeks gestation (19). These early thymocytes do not display the full range of T cell receptors (TCR) until 16 weeks GA (20). Fetal T cells can be detected in peripheral blood from 8 weeks GA, with the capacity to proliferate and produce cytokines (21). T cell migration to secondary lymphoid tissue has been confirmed with identification of T cells in the mesenteric lymph nodes (MLN) from 12 weeks GA (20) and the spleen and intestinal mucosa from 14 weeks GA (20–23).

Unsurprisingly, the majority of T cells derived from cord blood in newborns are naïve (24). The proportion of naive T cells has been shown to remain high (median 85%) at 6–8 weeks age [postnatal age (PNA)] in preterm infants, however this is significantly less than term infants at this PNA (25). This may reflect increased antigen exposure or reduced thymic output in preterm infants.

The size, composition, and function of the immune cell compartment varies from one tissue to another (26, 27). Interestingly, fetal CD4 T (T_h_) cells in the intestinal lamina propria but not spleen or liver display a predominantly memory phenotype (22, 23). This suggests priming as a result of prior “antigen” exposure, the nature of which is not known (22). Fetal MLN-derived T cells have capacity to respond to stimulation by proliferating and secreting a broad range of cytokines. Both proliferation and cytokine production by fetal MLN-derived T cells are augmented upon the removal of T_reg_s (20) suggesting T_reg_s play an important role in limiting fetal tissue inflammation.

## Skewing of the T-helper (T_h_) Response

Naïve T_h_ cells can differentiate toward alternative cell fates depending on the context in which they receive antigenic stimulation (8). Preterm infants have been suggested to have a skewed T_h_2 response based on their increased production of a classical T_h_2 cytokine, IL-5, and decreased production of a T_h_1 cytokine, IFN-γ, upon stimulation of peripheral blood compared to term cord blood (7). Postnatally, the ability of preterm T_h_ cells to secrete IFN-γ remains low with single cell RNA-seq analysis of term and preterm infants at 12 weeks of age demonstrating up-regulation of genes that suppress IFN-γ, together with longitudinal sampling of preterm infants displaying low expression (6, 13). An effective T_h_1 response is key to preventing intracellular infections, including bacteria, and this may explain why preterm infants have increased susceptibility.

In contrast to the T_h_2 skewing observed in peripheral blood, fetal intestinal T_h_ cells instead have a tendency to secrete TNF-α and IL-2 (T_h_1 cytokine), when compared to term infant intestinal samples. TNF-α was shown in a human fetal organoid model to be important in intestinal epithelial growth by its effects on intestinal stem cells, albeit high levels of TNF-α suppress epithelial growth. Infants with NEC have an increased production of TNF-α from intestinal T_h_ cells, although the temporal relationship to disease is unknown (23).

The tissue T_h_ response is affected by human milk oligosaccharides (HMO's), which comprise 1–2% of human milk and are the third largest non-water component by weight (after lactose and lipids). HMO's are not digestible by the infant but are believed to modulate the gut microbiota. HMO's appear to promote the growth of *Bifidobacteria* and suppress potentially pathogenic organisms (28, 29). When adding HMO's to fetal *in vitro* organ cultures, gene transcripts associated with T_h_ differentiation were found only with HMO's from early, not mature human milk, believed to reflect variation in the concentration of the HMO's. These transcriptional changes were deduced to promote a T_h_1 response whilst suppressing a T_h_2, T_h_17, and IL-8 expression (30). It is possible that these alterations play a role in the reduction of NEC and LOS seen in preterm infants fed breast milk.

## Suppression of the Immune Response

T_reg_s are a population of T_h_ cells defined by their suppressive function toward effector T cell responses. T_reg_ abundance is inversely correlated with GA and higher in infants compared to adults (9, 31). T_reg_s have been identified in the thymus as early as 13 weeks GA and in the periphery (spleen) from 14 weeks GA (21). The proportion of T_reg_s in the fetal MLN is significantly increased compared to adults and T_reg_s are functional even at a fetal stage (20, 23).

Interleukin- (IL-) 10 is amongst the factors that mediate the suppressive function as well as induce expansion of T_reg_s (8–10). IL-10 is produced following bacterial invasion or in stimulated tissues. IL-10 has been shown to contribute to bacterial clearance, yet minimize host damage from infection (10). Interestingly, germline mutations causing loss of function in the IL-10 receptor, as well as deficiency of the critical T_reg_ transcription factor *FOXP3*, cause early-onset enterocolitis (32). The ability of lamina propria T_reg_s to produce IL-10 is greatly reduced in preterm infants with NEC compared to term infants (23). An impaired T_reg_ IL-10 response to bacterial invasion may have a role in development of NEC, or it may be that the microbial dysbiosis seen before the development of NEC leads to an impaired IL-10 response (2).

Similar to humans, mice harbor an increased density of T_reg_s in the intestine compared with other organs. In mice, the introduction of gut bacteria induces an increase in the population of colonic but not small intestinal T_reg_s, apparently driven by *Clostridium*. These T_reg_s are thought to be peripherally induced (rather than thymus-derived) as judged by their lack of expression of the transcription factor, Helios (33). *Bacteroides fragilis* has also been shown to induce T_reg_s in mice through its production of polysaccharide A (PSA) in the presence of the toll-like receptor, TLR-2 (34, 35). In mice, induction of T_reg_s upon treatment by PSA in the presence of *Bacteroides fragilis* is protective against not only intestinal inflammation but also encephalitis, suggesting there could be a systemic effect of this interaction (34–36). No preterm human work on this aspect currently exists.

## Unimpaired CD8 Effector Potential?

CD8 T cells have been demonstrated in the fetal intestine as early as 16 weeks GA (20, 37). Whilst they display mainly a naive phenotype in the first 12 weeks of postnatal life, little is known about their postnatal effector potential (11). Fetal (term and preterm cord blood) and adult CD8 T cells develop a similar memory phenotype and ability to produce perforin and cytokines in response to a common neonatal virus, cytomegalovirus (CMV) (38). Furthermore, using cord blood derived CD8 T cells from varying gestations (23–41 weeks GA), an increased ability to secrete IFN-γ, TNF-α, and IL-2 has been demonstrated at earlier gestations (12).

These limited data suggest prematurity does not prevent CD8 effector T cell function in newborns. Indeed, it is possible that they display an excessive response to viral infection, which may be harmful, particularly as many infants are postnatally exposed to CMV.

## Innate-Like T cells

Innate-like T cells can display effector function without prior antigen priming. This would suggest they could be important in early life (17, 39). Innate-like T cells include γδ T, iNKT, and MAIT cells.

### Gamma-Delta (γδ) T Cells

Unlike αβ T cells, a large number of γδ T cells reside in non-lymphoid tissues including the gut, spleen and lungs. They have a small range of antigen receptors but are able to respond to a large repertoire of antigens including peptide not bound to classical major histocompatibility complex (MHC) molecules (39). They comprise 4–10%, 1–3%, and <1% of T cells in the peripheral blood of adults, term and preterm cord blood of infants, respectively (14).

γδ T cells develop a memory phenotype in the first month of life (15) and preterm γδ T cells have an increased ability to secrete IFN-γ and IL-10 upon stimulation with PMA and ionomycin compared to both term γδ T cells and preterm αβ T cells (15). The ability to secrete IFN-γ increases over time, which is in contrast to preterm T_h_ cells (13). However, when challenged with influenza virus, cord blood γδ T cells show a decreased ability to proliferate and produce IFN-γ compared to term infants and adults (14). The differences in these results could be due to experimental design, however these data suggest at least the potential for preterm γδ T cells to contribute to the cellular immune response. Little is known about preterm mucosal γδ T cells or their responses to alternative ligands.

### Invariant Natural Killer T (iNKT) Cells

Natural killer T (NKT) cells define a population of T cells that bear TCR's restricted by a non-classical MHC molecule, CD1d, and express cell surface markers associated with NK cells. Invariant/ type 1 NKT (or iNKT) cells express the invariant TCR (Vα24-Jα18). iNKT cells vary in abundance depending on tissue, comprising 0.01–0.1% of lymphocytes in peripheral blood but 10% of lymphocytes in the omentum (40). There is an increased abundance of iNKT cells in preterm cord blood compared to term cord blood (16).

iNKT's are able to secrete cytokines that mediate T_h_1, T_h_2, or T_h_17 responses, and recognize specific glycolipid antigens presented by CD1d (41). The ability of preterm iNKT cells to produce these T_h_ cytokines is not known but has been demonstrated in the case of term cord blood iNKT's. The secretion of IL-10 is particularly increased compared to adults suggesting a potential regulatory role of iNKT's in term infants (41).

iNKT cells have been associated with colitis in mouse models. Germ-free mice have an increased proportion of iNKT cells in their colon and increased susceptibility to colitis, however colonization of the intestine with bacteria early in life leads to a reduction in iNKT density and provides protection from colitis (42). iNKT cells able to produce IL-13 have been implicated in the pathogenesis of colitis (43). Furthermore, iNKT-deficient mice differ in gut microbial composition compared to wild-type mice, as well as showing increased intestinal leucocyte infiltration (44). Whether iNKT cells are involved in the pathogenesis of NEC in preterm infants is not known.

### Mucosa-Associated Invariant T (MAIT) Cells

MAIT cells are a population of T cells predominantly found in the lung and intestinal mucosa, that display a semi-invariant TCR (Vα 7.2 – Jα3.3/20/12). MAIT cells have been demonstrated in the fetal thymus from 18 weeks GA. MAIT cells represent <1% of peripheral blood T cells in newborns compared to up to 10% in adults, suggesting a postnatal expansion (17). MAIT cells are of particular interest as they recognize MR-1, an MHC-1 like molecule that presents microbial derived metabolites of the essential vitamin, riboflavin (17). Riboflavin is synthesized by gut commensal bacteria, and has a variable concentration in human milk dependent on maternal intake (45, 46). Thus, the postnatal expansion of MAIT cells is likely driven both by bacteria and by diet.

In contrast to thymic MAIT cells, those in fetal intestine, spleen, and MLN express a marker of activation (PLZF), suggesting peripheral maturation. They develop this mature phenotype in the first 2 months of life (17, 47) and display inflammatory and cytotoxic abilities. Indeed, fetal intestinal, liver and lung but not thymic or spleen MAIT cells can produce IFN-γ following stimulation (18). Functional changes in MAIT cells have been associated with inflammation in adults with inflammatory bowel disease (48). However, no study has examined their relationship with NEC.

## Dietary Interactions With Mucosal T Cells

**Figure 1:**
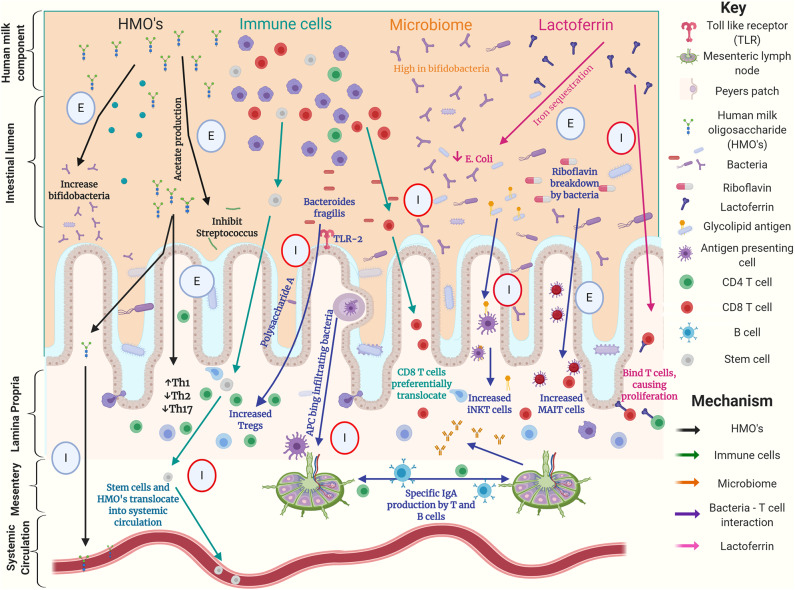
Graphical summary of possible interactions between components of human milk, the gut microbiome and T cells in the preterm intestine. This summarizes work using human, animal and laboratory-based experiments. Circles highlight experiment type with; E denoting an *ex-vivo* experiment, I denoting an *in-vivo* experiment, red circles denoting animal experiments (16, 28–30, 32, 34, 35, 42, 44, 49–57).

When considering modulation of the preterm T cell populations, we have described how key dietary exposures such as HMOs and bacteria appear important. Breast milk contains a vast array of components that may directly or indirectly affect gut mucosal T cell populations, as demonstrated in both human and animal models of early life (Figure 1). The gut microbiota is likely to be a key mediator of such dietary effects, implying the possibility of therapeutic modulation either by dietary modifications or the use of prebiotics or probiotics.

### Colostrum

Compared to more mature human milk, colostrum has a higher concentration of bioactive components including HMOs, lactoferrin, immune cells, and immunoglobulin. In a porcine model, pregnant sows were given NEC-inducing intra-amniotic lipopolysaccharide injections *in-utero* and offspring pups were then fed varying diets before euthanasia. Pups fed colostrum had decreased levels of distal ileal IL-8 and IL-1β, combined with increased abundance of blood T_h_ cells (as % of lymphocytes). The authors concluded that a colostrum diet led to maturation of the intestinal mucosa as well as the systemic immune system (50).

### Translocation of Milk Derived Immune Cells

Human milk contains stem cells and leucocytes (51, 58). Studies using mice show that maternal immune cells can translocate from the intestine into the circulation or distal organs (53, 54, 59). Stem cells derived from milk have been shown to translocate into the brains of mice, and once there to traffic and differentiate (53, 54). In these mouse models, T cells are the predominant cell type that transfers across the intestinal epithelium despite their relative paucity in milk (58, 59). CD8 T cells, in particular, translocate into the intestinal Peyer's patches, possibly based on their expression of a gut homing receptor (CCR9). Once there, they show an increased ability to produce cytokines upon stimulation compared with peripheral blood CD8 T cells (59). It is unclear whether diet-derived T cells migrate beyond the MLN in mice as studies conflict (52, 59) but it is plausible that milk-derived maternal CD8 T cells translocate into the mucosa in humans and compensate for “deficiencies” of preterm T cells.

### Lactoferrin

Lactoferrin is the major whey glycoprotein of human milk and subject to intense research for over 50 years due to its ability to inhibit bacteria by multiple mechanisms (55, 60). Lactoferrin has also been shown to bind to immune cells and influence their function (61). Most lymphocytes can express a lactoferrin receptor including stimulated αβ and γδ T cells (56). Furthermore, T cells in the lamina propria of pigs bind lactoferrin (57). In a mouse model, T cells in the lamina propria proliferated upon administration of enteral bovine lactoferrin in the presence or absence of colon cancer. These T cells secreted IFN-γ and IL-18, suggesting that they were immunologically active (62).

## Conclusion

Preterm infants have a T cell population that was designed for fetal life and therefore differs systematically from term infants (Table 1). In particular, they have a diminished T_h_1 response leaving them susceptible to infection by intracellular pathogens. T_reg_s protect the preterm infant from an excessive innate response which may reduce the risk of NEC but may also increase the risk of LOS. This variation in function of T_reg_s is modulated by IL-10.

There are multiple mechanisms by which the diet, gut microbiome, and T cell populations have been demonstrated to interact in human, mouse, and laboratory experiments demonstrated in Figure 1. However, there is a paucity of data comparing the effects of alternative feeding strategies on the preterm infant's immune system, including T cells, and there remains substantial uncertainty about when and how donor human milk and human milk derived fortifiers should be used. Clinical studies aimed at identifying changes in the immune system associated with these dietary interventions will improve understanding and enable more informed nutritional management.

The functions of innate-like cells such as iNKT and MAIT cells in preterm infants have yet to be unraveled but a number of compelling studies on animals imply an important role in preventing intestinal inflammation. Alterations in gut mucosal T cells may occur in preterm infants as a result of dietary or microbiome manipulations. A better understanding of the interplay of diet, microbiome and host immunity will underpin efforts to develop interventions that modulate T-cell responses for improved infant health.

## Author Contributions

TS wrote the initial manuscript. All authors contributed to manuscript revision, read, and approved the submitted version.

## Conflict of Interest

NE declares research funding from Danone Early Life Nutrition and Prolacta Biosciences, as well as speaker honoraria from Baxter and Nestle Nutrition Institute. The remaining authors declare that the research was conducted in the absence of any commercial or financial relationships that could be construed as a potential conflict of interest. The handling editor declared a past co-authorship with the JB and NE.

## Abbreviations

GA: gestational age
iNK T: invariant natural killer T cells
MAIT: mucosal associated invariant T cells
MHC: major histocompatibility complex
MLN: mesenteric lymph nodes
PNA: postnatal age
TCR: T cell receptor
T_h_: T-helper
T_reg_s: regulatory T cells.
